# Dimensional Control in Phase-Pure Coevaporated Quasi-2D Ruddlesden–Popper Structures

**DOI:** 10.1021/jacs.4c18641

**Published:** 2025-04-29

**Authors:** Kunal Datta, Pranav Khadilkar, Honghu Zhang, Diana K. LaFollette, Esteban Rojas-Gatjens, Ruipeng Li, Guoxiang Hu, Juan-Pablo Correa-Baena

**Affiliations:** 1 School of Materials Science and Engineering, Georgia Institute of Technology, Atlanta, Georgia 30332, United States of America; 2 National Synchrotron Light Source II, Brookhaven National Laboratory, Upton, New York 11973, United States of America; 3 School of Chemistry and Biochemistry, Georgia Institute of Technology, Atlanta, Georgia 30332, United States of America

## Abstract

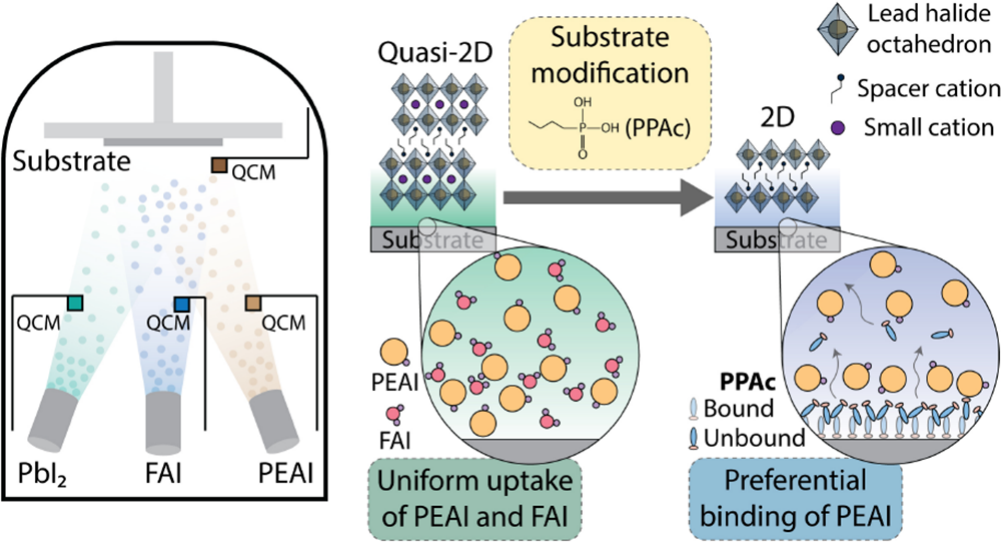

Fast, uncontrolled crystallization with several competing pathways makes solution-processing of phase-pure quasi-two-dimensional (quasi-2D) metal halide Ruddlesden–Popper thin films challenging. Typically, solution-processing results in the formation of different structural phases with varying dimensionality ranging from 2D, to quasi-2D, and 3D, introducing bandgap disorder and inhibiting charge transport. In this work, we eliminate interactions between precursor salts and solvents by using controlled thermal coevaporation to grow quasi-2D thin films that show high phase purity and narrow phase distribution. We study the structural landscape using synchrotron-based X-ray scattering and charge-carrier dynamics using ultrafast pump–probe spectroscopy. We then demonstrate a strategy to control the crystallographic phase of the film through phosphonic acid-based surface modification. We use density functional theory to study the interactions between propylphosphonic acid and the organic precursors and find that the interactions of loosely bound phosphonic acid molecules with evaporated precursors, followed by the migration of phosphonic acids through the deposited thin film, dictate the film structure between 2D and quasi-2D phases. These findings introduce new solvent-free methods for the fabrication of phase-pure quasi-2D Ruddlesden–Popper thin films and control phase selectivity across different dimensional (2D and quasi-2D) structures.

## Introduction

Ruddlesden–Popper structures are lower-dimensional analogs of three-dimensional (3D) perovskites formed by sheets of lead iodide octahedra and small cation networks interspersed with large organic spacer cations. These structures are identified by an *n*-value that indicates the number of conjoined sheets of lead halide octahedra (*n* = 1 is a two-dimensional (2D) structure, and *n* = ∞ refers to a 3D structure), and modifications to the crystalline structure by changing the organic spacer cation or altering the dimensionality (*n*-value) influence the optical bandgap, exciton binding energy, defect formation and migration energy, and charge-carrier mobility.^1−4^ In particular, quasi-2D structures that are intermediate to 2D and 3D phases present advantages such as stability against ion migration and environmental stressors while allowing high charge conductivity, making them suitable for a variety of optoelectronic applications.^4−6^

However, commonly used solution-based processing methods to coat quasi-2D thin films present inherent disadvantages that limit the film quality and charge-carrier dynamics. Here, interactions between precursor salts, such as phenethylammonium (PEA^+^) iodide, butylammonium iodide, methylammonium iodide, and formamidinium (FA^+^) iodide, and commonly used solvents such as *N*,*N*-dimethylformamide (DMF) and dimethyl sulfoxide trigger complex crystallization processes that result in the formation of a distribution of dimensional structures.^5,7^ Solution-processed films therefore develop structural/compositional gradients across the thickness of the film resulting from the growth of disordered layers with several coexisting phases with different dimensionality.^2,8,9^ Issues around solvent orthogonality also prevent the deposition of heterostructured thin films through solution-processing routes where the thickness of different dimensional phases can be precisely controlled.^10^ Overall, these constraints severely narrow the processing window for lower-dimensional quasi-2D perovskites, restricting broad applicability in devices.

Thermal coevaporation is a promising fabrication route to deposit quasi-2D films with higher phase purity. The method eliminates precursor–solvent interactions and allows the growth of structurally homogeneous layers with high thickness control.^11^ Other advantages such as conformal deposition, compatibility with large-scale processing, and elimination of toxic solvents also make the technique relevant for upscaling Ruddlesden–Popper-based optoelectronic device platforms.^12−14^ Physical vapor deposition has already been successfully used to process 3D perovskite-based devices and is being developed for growing lower-dimensional structures.^15−17^ For example, organic spacer cations have been sublimed onto 3D perovskite layers to form Ruddlesden–Popper interfacial structures.^18−21^ We have also recently reported the coevaporation of 2D Ruddlesden–Popper interfacial layers for solar cells.^22^ Other physical vapor deposition methods, such as single-source pulsed laser deposition, have also been used to coat 2D perovskite films.^23^ Furthermore, attempts have been made to coat quasi-2D films, either through two-step physical vapor deposition or through single-source evaporation of ground quasi-2D crystals.^24−26^

In this work, we study the crystallization of quasi-2D Ruddlesden–Popper thin films grown using thermal coevaporation. We use PEA^+^ as the organic spacer cation and FA^+^ as the small organic cation to grow *n* = 2 quasi-2D structures with significantly higher phase purity compared with solution-processed films. We use phosphonic acid-based substrate functionalization to modulate phase distribution in coevaporated quasi-2D films. The strategy is guided by the interaction of phosphonic acid with PEA^+^ and FA^+^, which controls the relative uptake of organic precursors and promotes the crystallization of different structural phases. We characterize the evolution of structural properties using synchrotron-based grazing incidence wide-angle X-ray scattering (GIWAXS) and its impact on charge-carrier dynamics using ultrafast pump–probe spectroscopy. The observations show that the use of phosphonic acid-based surface modification favors the formation of lower-dimensional structures, allows a narrower dimensional distribution, and enables the formation of 2D/quasi-2D Ruddlesden–Popper heterostructures. Our work advances solvent-free coating methods to grow quasi-2D Ruddlesden–Popper films with increased phase purity and demonstrates strategies for phase tunability in such films.

## Results and Discussion

**Figure 1:**
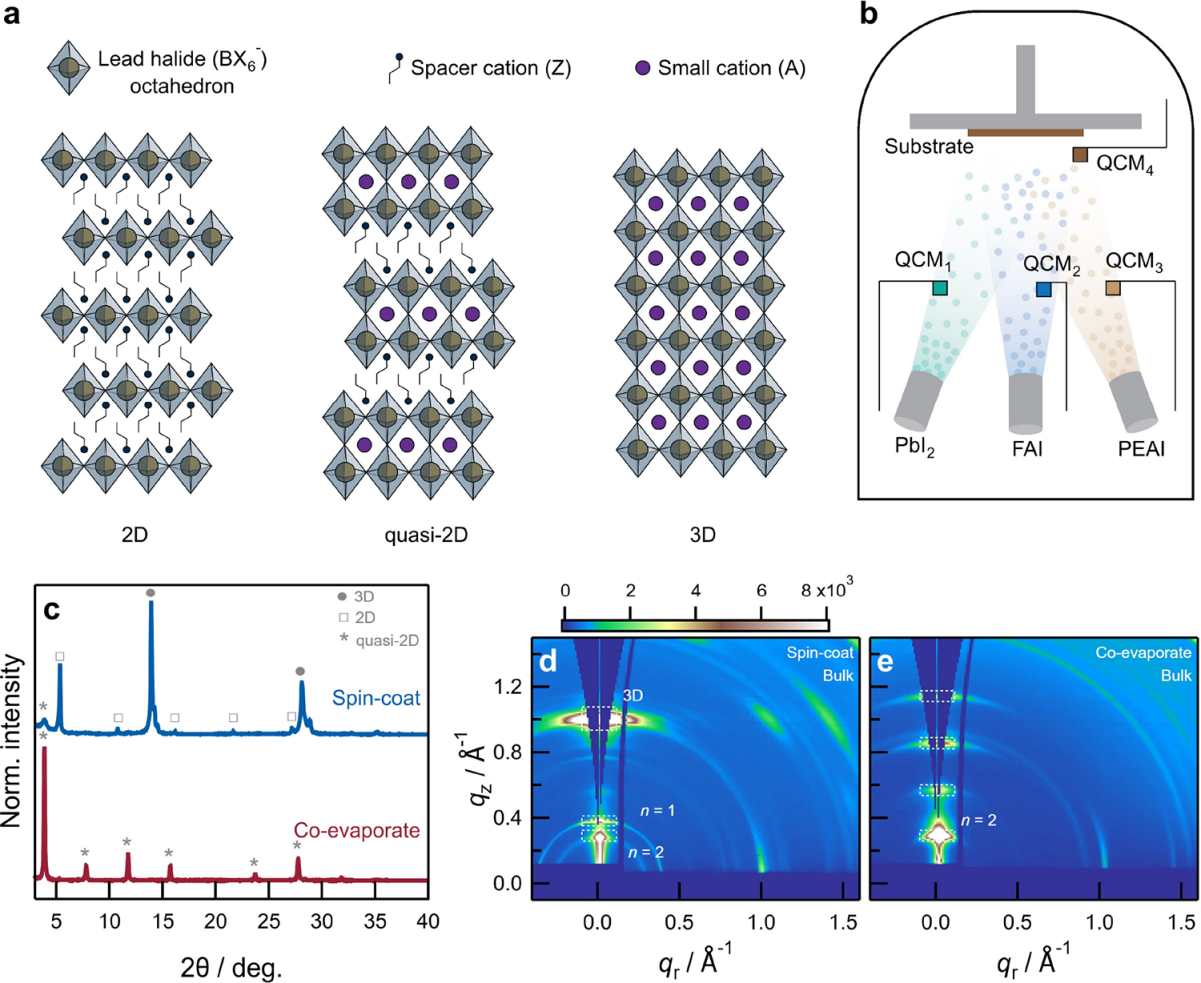
(a) Structures of Ruddlesden–Popper structures with *n* = 1 (2D), *n* = 2 (quasi-2D), and *n* = ∞ (3D). (b) Schematic of the thermal coevaporation deposition method. (c) XRD pattern of the spin-coated and coevaporated Ruddlesden–Popper thin films (nominally PEA_2_FAPb_2_I_7_) with *n* = 2 deposited on ITO-coated glass substrates. Synchrotron-based GIWAXS patterns of Ruddlesden–Popper thin films with *n* = 2 prepared through (d) spin-coating and (e) coevaporation. Dashed rectangles highlight scattering features related to specific structural phases in panels (d) and (e).

Figure 1a shows the schematics of *n* = 1 (2D), *n* = 2 (quasi-2D), and *n* = ∞ (3D) Ruddlesden–Popper structures with a nominal composition of *Z*_2_*A*_*n*–1_*B*_*n*_*X*_3*n*+1_ for the lower-dimensional (2D and quasi-2D) structures. Here, *Z* represents spacer cations such as PEA^+^, *A* represents small organic cations such as FA^+^, *B* is a divalent cation such as Pb^2+^, and *X* is a monovalent anion such as I^–^. We deposited *n* = 2 PEA_2_FAPb_2_I_7_ thin films using spin-coating from a DMF-based solution and from a three-source thermal coevaporation method (Figure 1b) where we independently monitored the evaporation of the FAI, PEAI, and PbI_2_ precursors using quartz crystal microbalances (see Supporting Information for details). The X-ray diffraction (XRD) patterns of the spin-coated films indicate the presence of several structural phases (Figure 1c). For example, the peak at 2θ ∼5.3° corresponds to the *n* = 1 PEA_2_PbI_4_, the peak at 2θ ∼3.9° corresponds to the *n* = 2 PEA_2_FAPb_2_I_7_ structure, and the peak at 2θ ∼13.9° corresponds to the 3D FAPbI_3_ perovskite.^22,27−29^ Additional Ruddlesden–Popper phases with *n* > 2 may also be present in the films with diffraction peaks at similar 2θ angles as the 3D phase.^30,31^ The observation of different dimensional phases is consistent with previous reports and poses a key challenge to the deposition of high-quality quasi-2D thin films. In contrast, the coevaporated film only shows diffraction peaks related to the *n* = 2 phase (2θ ∼ 3.9°), demonstrating the higher phase purity in coevaporated films compared to spin-coated films.

Synchrotron-based GIWAXS was used to study the structural characteristics of the thin films (Figures 1d,e). The spin-coated film shows features corresponding to the presence of *n* = 1 (*q* ∼ 0.37 Å^–1^) and *n* = 2 (*q* ∼ 0.28 Å^–1^) Ruddlesden–Popper phases as well as the formation of the 3D phase (*q* ∼ 0.99 Å^–1^).^2,32−34^ A strong orientation of the feature at *q* ∼ 0.99 Å^–1^ also indicates the possible presence of other higher-dimensional Ruddlesden–Popper phases (*n* > 2).^35^ Coevaporated films show a singular preferentially oriented feature corresponding to the *n* = 2 phase (*q* ∼ 0.28 Å^–1^). It is important to note that the coevaporated film undergoes no postdeposition treatment, such as thermal annealing, emphasizing the high quality of the film achieved by vapor-based processing. We varied the angle of incidence (α) of the X-ray beam between 0.05° and 0.5° (Figure S1) to study the surface and bulk structural properties of thin films, respectively.^36^ In a spin-coated film, the surface structure (α = 0.05°) is predominantly in the 3D phase with a minor contribution from lower-dimensional (*n* = 1 and *n* = 2) structures. With an increasing incidence angle, probing deeper in the film, the relative contribution of the 2D phase increases with respect to the 3D phase. This indicates the presence of a structural gradient wherein the film is richer in the 2D phase as a function of depth.^5^ The observation agrees with prior work that shows the formation of “regular” dimensional gradients in quasi-2D perovskite films where strong interactions between spacer cations and solvents cause the surface crystallization of 3D phases followed by crystallization of lower-dimensional phases at the interface with the substrate.^5,7−9^

In contrast, coevaporated films show minimal differences in phase behavior as a function of film depth, primarily showing features at *q* ∼ 0.28 Å^–1^, corresponding to the *n* = 2 phase. This indicates the formation of a homogeneous film with no significant structural gradients in coevaporated films. We also tracked the structure of the coevaporated thin film as a function of exposure to oxygen and humidity (Figure S2, Supporting Information) and found the film to be structurally stable over long exposure.

Surface modification strategies can be used to control crystallization processes in halide perovskite thin films.^37,38^ For example, phosphonic acids have been used as surface modifiers or as charge transport/injection layers in devices.^30−32,38^ They have also been shown to influence phase distribution in Ruddlesden–Popper quasi-2D structures.^35,39^ In coevaporation deposition of 3D perovskites, phosphonic acids have recently been shown to increase the uptake of organic precursors.^29,40^ Furthermore, loosely bound excess phosphonic acids on the substrate surface can also migrate through the film and progressively participate in crystal nucleation as the perovskite film grows.^29^ We studied the interactions between propylphosphonic acid (PPAc) and organic species (PEA^+^ and FA^+^) in coevaporated films. Here, unlike the case of 3D perovskite crystallization,^29^ however, PPAc substrate modification agents can interact with both PEA^+^ and FA^+^ during the crystallization of coevaporated quasi-2D films.

**Figure 2:**
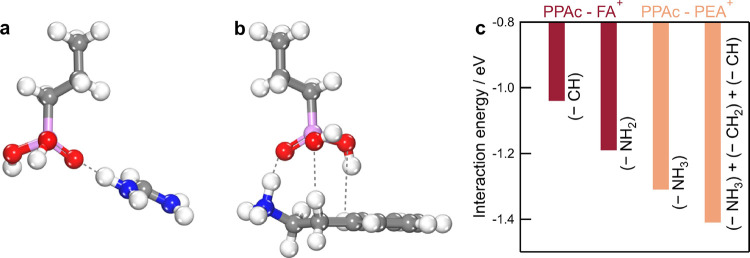
Optimized atomic structures of interactions between (a) PPAc and FA^+^ and between (b) PPAc and PEA^+^. (c) Interaction energies of different hydrogen bonding modes between PPAc and FA^+^ (red) and between PPAc and PEA^+^ (orange). Carbon, hydrogen, phosphorus, oxygen, and nitrogen atoms in panels (a) and (b) are indicated by gray, white, pink, red, and blue spheres, respectively, and dashed lines indicate different hydrogen bonding interactions.

We performed density functional theory (DFT) calculations to understand the interaction of PPAc with the two organic precursors. PPAc-FA^+^ interaction occurs through hydrogen bonding between the oxygen atom in PPAc and either the −CH or the −NH_2_ moieties in FA^+^. The interaction energies were found to be −1.04 and −1.19 eV, respectively. Figure 2a shows the optimized atomic structure for the interaction between PPAc and the −NH_2_ moiety in FA^+^. On the other hand, three hydrogen bonding modes were investigated for PPAc-PEA^+^ interaction: (1) between the −CH moiety in the aromatic ring and PPAc, (2) between the −CH_2_ moiety in the alkyl chain and PPAc, and (3) between the terminal −NH_3_ moiety in PEA and PPAc. Among them, the interaction between the terminal −NH_3_ moiety and PPAc was found to be the most energetically favorable, with an interaction energy of −1.31 eV (Figure 2b,c). Furthermore, the interaction energy can be increased to −1.41 eV by the presence of additional interaction sites on the aromatic ring and alkyl chain (Figure 2b,c). Therefore, based on these calculations, we conclude that the interaction between PPAc and PEA^+^ is significantly stronger than between PPAc and FA^+^, implying that the relative uptake of PEAI in the coevaporated film will likely increase in the presence of PPAc.

**Figure 3:**
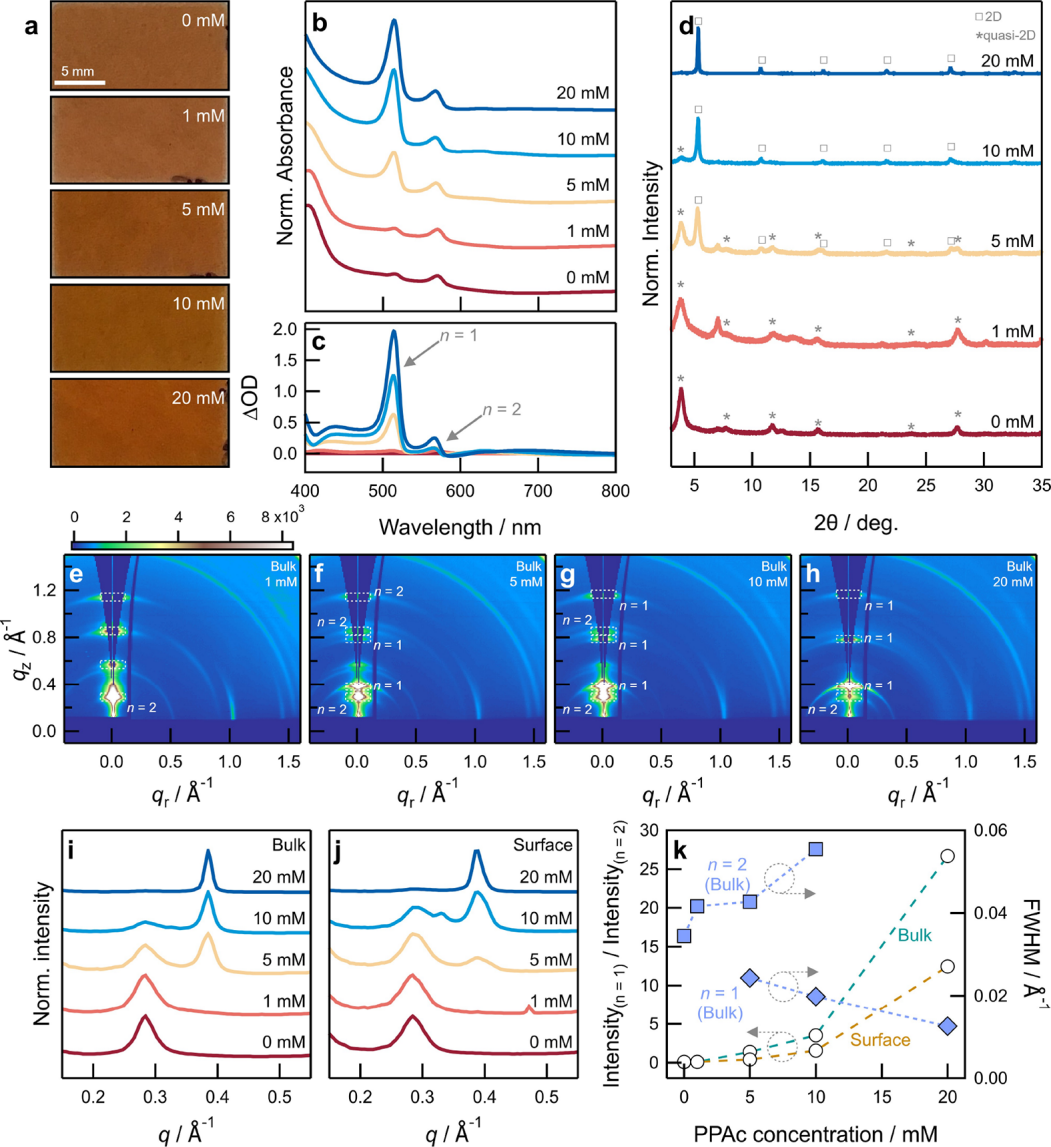
(a) Images of coevaporated Ruddlesden–Popper films on PPAc-functionalized substrates with different PPAc concentrations. Scale bar is 5 mm. (b) UV–vis–NIR spectra of coevaporated Ruddlesden–Popper films. Spectra are normalized to the intensity of the excitonic feature at λ = 571 nm. (c) Corresponding differential absorption spectra calculated by subtracting the absorption (optical density) of the control coevaporated film (0 mM) from unnormalized absorption spectra of coevaporate films on PPAc-functionalized substrates. (d) XRD patterns of coevaporated Ruddlesden–Popper films on PPAc-functionalized substrates with different PPAc concentrations. (e–h) 2D GIWAXS patterns of coevaporated Ruddlesden–Popper films acquired with α = 0.5° (Bulk). (i) Sector average of GIWAXS patterns in panels (e–h) acquired with α = 0.5°. (j) Sector average of GIWAXS patterns acquired with α = 0.05° (surface). (k) Intensity ratio (left *y*-axis) of *n* = 1 and *n* = 2 diffraction features acquired from sector averages at α = 0.05° (surface, ochre) and α = 0.5° (bulk, teal), and full width at half-maximum (fwhm) (right *y*-axis, blue) of *n* = 1 and *n* = 2 diffraction features acquired from sector averages as a function of PPAc concentration. Film thickness is 150 nm.

We spin-coated PPAc on ITO-coated glass substrates from ethanol-based precursor solutions with increasing concentrations (0 to 20 mM). Increasing the precursor solution concentration increases the concentration of loosely bound PPAc molecules available on the substrate surface after the active sites on ITO have been saturated.^29^ We then coevaporated 150 nm *n* = 2 quasi-2D Ruddlesden–Popper films on the functionalized substrates and characterized their optical and structural properties (Figure 3). The films darken with an increasing PPAc concentration on the substrate (Figure 3a). On a bare ITO surface (0 mM PPAc), the ultraviolet–visible–near-infrared (UV–vis–NIR) spectrum shows an excitonic peak at ∼571 nm corresponding to the *n* = 2 phase (Figure 3b).^28^ A shallow and broad absorption onset at ∼663 nm and a minor excitonic peak at ∼517 nm, however, indicate the presence of undesired minority phases with higher (*n* > 2) and lower (*n* = 1) dimensionality, respectively. With increasing PPAc concentration in the precursor solution, two changes are observed in the absorption spectra of coevaporated films. First, the absorption feature related to an *n* = 1 phase (λ ∼ 517 nm) increases significantly (Figures 3b,c) indicating an increased volume fraction of the *n* = 1 phase over the *n* = 2 phase in the film. Second, the low-energy absorption feature shows a concurrent decrease and is absent at a PPAc concentration of 20 mM (Figure 3b). The film structure shows similar changes wherein with increasing PPAc concentration (Figure 3d), the intensity of the diffraction peak corresponding to the *n* = 2 phase (2θ ∼ 3.9°) undergoes a relative decrease while the intensity of the diffraction feature related to the *n* = 1 phase (2θ ∼ 5.3°) concurrently increases. At high PPAc content (20 mM), the diffraction peak related to the *n* = 2 phase is absent and the structure is dominated by the *n* = 1 phase. Based on the optical and structural observations, it can therefore be argued that the impact of PPAc on film crystallization shifts the phase distribution toward lower *n*-value Ruddlesden–Popper structures.

We then characterized film structure as a function of depth using synchrotron-based angle-resolved GIWAXS. The 2D patterns of the bulk structure (α = 0.5°) show that at low PPAc concentration (<5 mM), the perovskite film is composed of the *n* = 2 phase (Figures 1e and 3e), indicated by a scattering feature at *q* ∼ 0.28 Å^–1^. With increasing PPAc concentration (5 and 10 mM); however, an additional peak at *q* ∼ 0.37 Å^–1^ appears, corresponding to the formation of the *n* = 1 phase (Figure 3f,g). Upon further increasing the PPAc concentration (20 mM), the *n* = 1 phase dominates the structure, and only a small proportion of the *n* = 2 phase is visible (Figure 3h). The sector average analysis (Figure S3, Supporting Information) shows this behavior clearly (Figure 3i) and that a progressive increase in PPAc concentration increases the relative proportion of the *n* = 1 phase in the bulk film compared to the *n* = 2 phase (Figure S4, Supporting Information).

We compared the structural properties in the bulk film (α = 0.5°) to the surface (α = 0.05°) (Figures S5 and S6, Supporting Information) and found similar trends wherein the relative proportion of the *n* = 1 phase increases with increasing PPAc concentration. However, this increase occurs more slowly in the surface structure than in the bulk structure as a function of PPAc concentration (Figure 3j). For example, at low PPAc concentrations (5 mM), the intensity ratio of the *n* = 1 and *n* = 2 scattering features () is 0.42 at the film surface and 1.38 in the film bulk (Figure 3k), indicating the bulk to be richer in the *n* = 1 phase than the surface.^5^ It must be noted that while the surface measurement samples the first few nanometers of the film, the bulk measurement samples both the surface and bulk regions of the film.^36,42^ Upon increasing the PPAc concentration (10 mM), this ratio increases to 1.59 at the film surface and 3.53 at the film bulk. Finally, at a PPAc concentration of 20 mM, the intensity ratio is 12.54 at the film surface and 26.68 in the film bulk.

The phase change (*n* = 2 to *n* = 1) in the bulk film as a function of the PPAc concentration is also accompanied by a concurrent change in crystallinity (Figure 3k). Here, with increasing PPAc concentration and consequent growth of the *n* = 1 phase, we find that the fwhm of the *n* = 2 diffraction peak increases from ∼0.034 to ∼0.055 Å^–1^ between PPAc concentrations of 0 and 10 mM. We attribute this decrease in crystallinity to an increase in disorder due to the formation of the additional *n* = 1 phase in the film.^43^ Similarly, the *n* = 1 phase is less crystalline when it is first detected in the multiphase (*n* = 1 and *n* = 2) film at PPAc concentration of 5 mM (fwhm ∼ 0.024 Å^–1^) but increases in crystallinity as it forms the dominant phase with increasing PPAc concentration, with fwhm ∼ 0.012 Å^–1^ at PPAc concentration of 20 mM. Finally, as PPAc impacts phase change, it also causes a concurrent increase in film thickness, with minimal change at low PPAc concentrations (≤5 mM), followed by a sharp increase of up to 70% at high PPAc concentration as the *n* = 1 phase starts to dominate the film structure (Figure S7, Supporting Information).^29^

**Figure 4:**
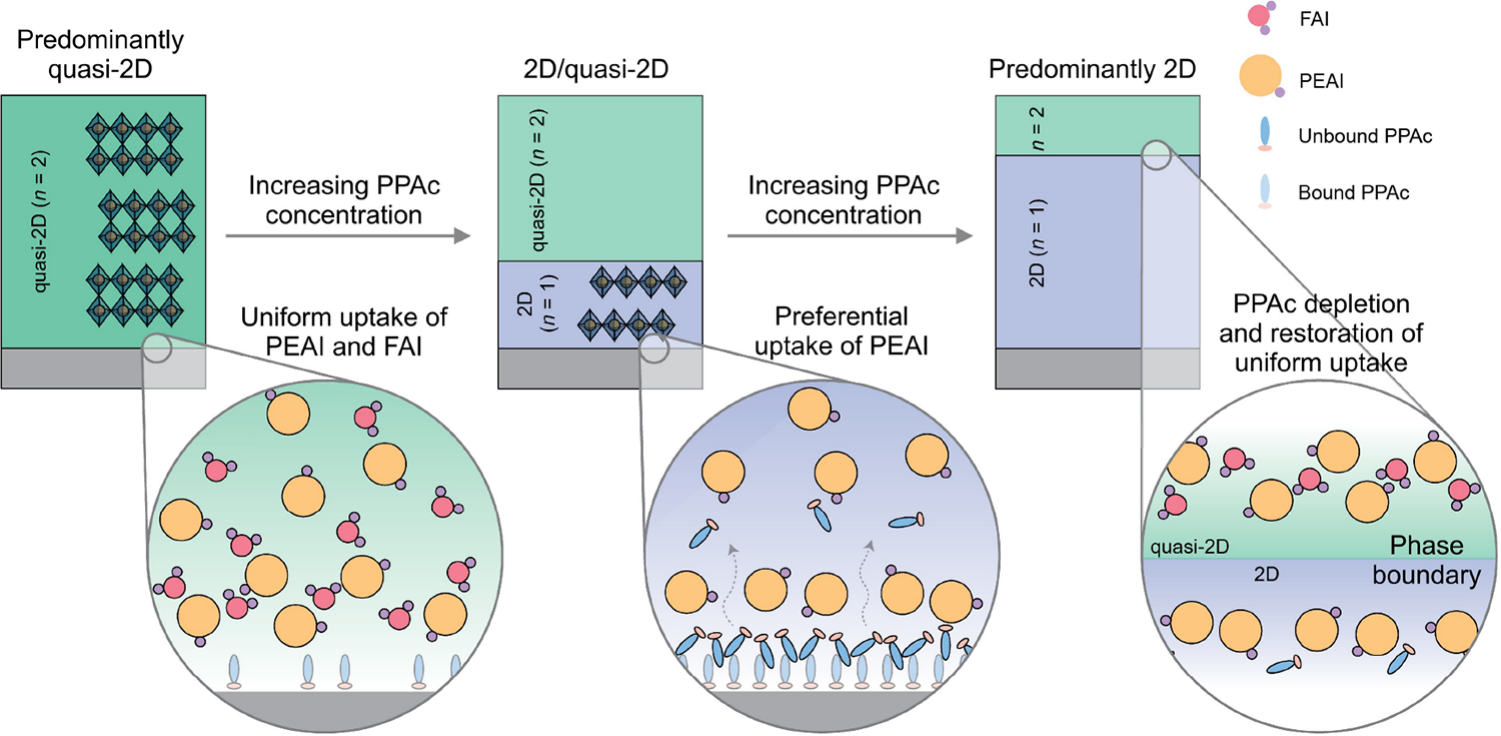
Crystallization mechanism of the Ruddlesden–Popper film on PPAc surface-modified substrates. The organic cations have been removed from the crystal structures of the Ruddlesden–Popper phases for clarity.

Taken together, we conclude that PPAc strongly impacts phase selectivity and crystallinity in coevaporated Ruddlesden–Popper films. In films where the *n* = 1 and *n* = 2 phases coexist, at PPAc concentration ≥5 mM, the bulk volumes near the interface with the metal oxide substrate preferentially crystallize as the *n* = 1 phase and the film surface form the *n* = 2 phase. This preferential crystallization is a consequence of the increased uptake of PEAI in the early stages of deposition due to its favorable interactions with PPAc that is concentrated at the substrate surface. At high PPAc concentrations, unbound PPAc molecules can additionally migrate through the deposited film and progressively nucleate the *n* = 1 phase in the bulk.^29^ The interaction and migration are related to the low intraligand interactions between PPAc molecules that allow it to interact with surrounding evaporated precursors and thin film.^29^ As PPAc diffuses and is depleted, the relative uptake of PEAI decreases, favoring the formation of the *n* = 2 phase close to the film surface. In such cases (PPAc concentrations 5 and 10 mM), the bulk structure shows stronger signatures of the *n* = 1 phase while the surface structure shows the presence of the *n* = 2 phase. As a result, the volume fraction of the *n* = 1 phase increases with increasing PPAc concentration, and at a high PPAc concentration (20 mM), the film is predominantly in the 2D phase (Figures 3h). We do not observe any anomalous changes to the lattice parameter, though, and therefore conclude that PPAc does not incorporate in the Ruddlesden–Popper structure but is likely embedded at grain boundaries. Altering the film microstructure, and thereby the grain boundary density, is therefore likely to impact the migration of PPAc. We describe this proposed mechanism in Figure 4.

We verified this hypothesis by coevaporating a thicker (300 nm) Ruddlesden–Popper film while varying the PPAc concentration (0–20 mM). Here, structural characterization (Figures S8 and S9, Supporting Information) shows that the appearance of the *n* = 1 phase occurs at PPAc concentration of 10 mM, compared to 5 mM for the 150 nm film, and increases thereafter at higher PPAc concentration. Comparing surface and bulk structures also shows that similar to thinner 150 nm films, the bulk structure has a higher proportion of the *n* = 1 2D phase while the surface is comparatively richer in the *n* = 2 quasi-2D phase (Figure S10, Supporting Information). These observations show that for the same PPAc concentration of 20 mM, a higher proportion of *n* = 2 phase is formed on the surface ( = 1.21) of a thicker quasi-2D film (Figure S10, Supporting Information), compared to a thinner film (Figure 3k,  = 0.08). This likely occurs due to the depletion of PPAc through the thickness of the 300 nm coevaporated film, which restricts the volume of the *n* = 1 phase closer to the substrate, whereas PPAc is able to migrate further through a 150 nm film to promote crystallization of the *n* = 1 phase.

**Figure 5:**
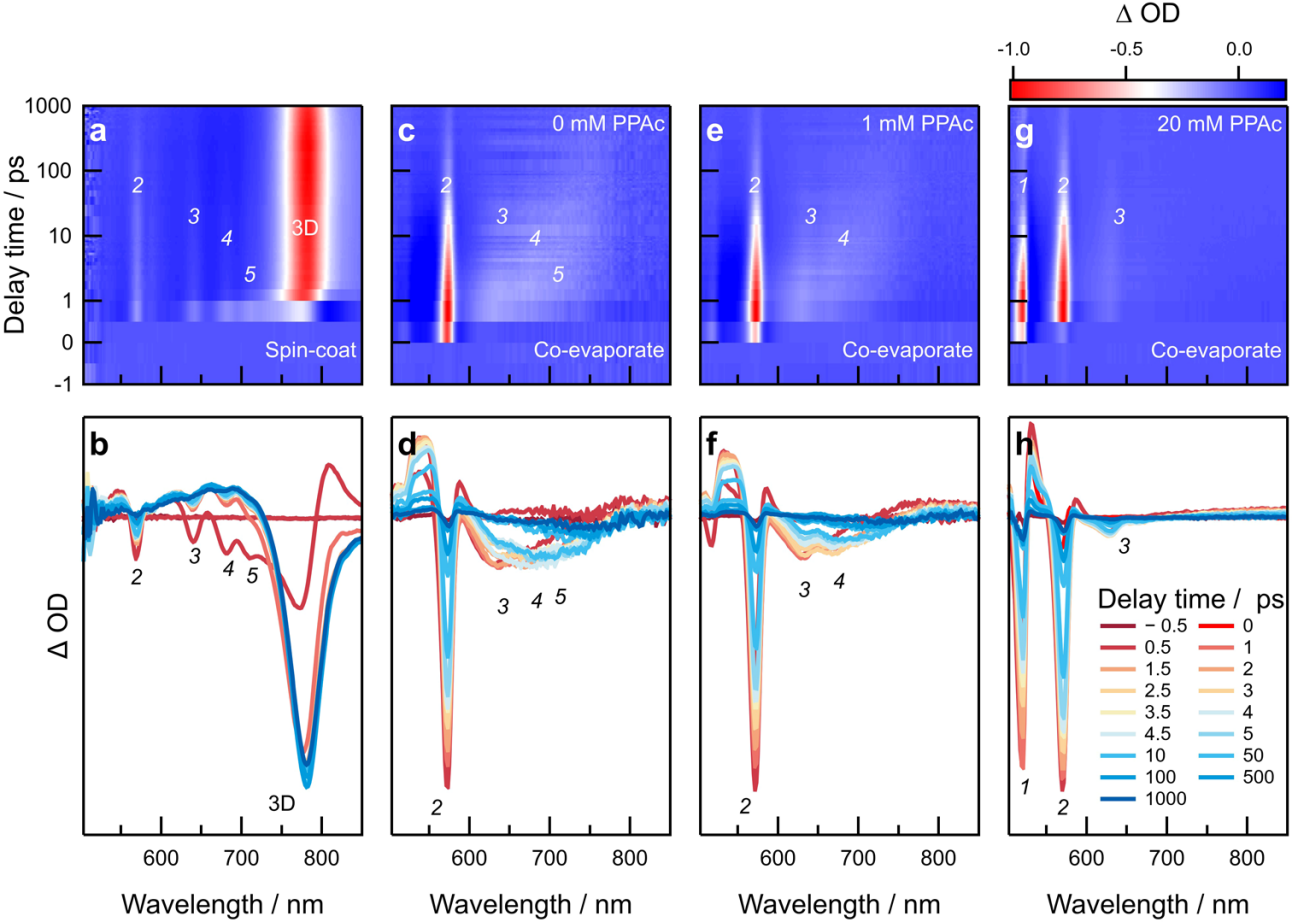
Transient absorption spectroscopy of spin-coated and coevaporated Ruddlesden–Popper films. 2D heatmaps and linecuts of (a,b) spin-coated film, (c,d) coevaporated film with 0 mM PPAc, (e,f) coevaporated film with 1 mM PPAc, and (g,h) coevaporated film with 20 mM PPAc. Note that the *y*-axis is plotted on the linear scale between −1 and 1 ps delay time and on the log scale between 1 and 1000 ps delay time in panels (a), (c), (e), and (g). The approximate positions of photobleaching features for different structural phases are indicated by the *n*-value or as “3D” in italics for clarity.

We studied charge-carrier dynamics in coevaporated Ruddlesden–Popper thin films using ultrafast pump–probe spectroscopy (Figure 5).^44^ A 470 nm pump was used to excite carriers in the thin films and probed with white light, incident on the sample after a time delay (Δ*t*), allowing probe of charge-carrier relaxation as well as charge transfer processes occurring in the film. As a result, in addition to being able to measure charge-carrier lifetimes, the technique also allows identifying secondary undesired photoactive species in the film.^2,7,39^ Secondary phases may often be poorly crystalline or not demonstrate preferential orientation, thereby proving hard to probe through structural characterization tools.^22,40^ For example, photobleaching features related to several lower-dimensional (*n* = 2 (λ ∼ 570 nm), *n* = 3 (λ ∼ 640 nm), *n* = 4 (λ ∼ 681 nm), *n* = 5 (λ ∼ 710 nm)) and 3D phases (λ ∼ 781 nm) are visible in the spin-coated PEA_2_FAPb_2_I_7_ film (Figure 5a).^34^ This observation agrees with the structural characterization that showed a mixture of Ruddlesden–Popper phases and a significant proportion of the 3D phase in spin-coated films (Figure 1c,d). However, the spectrum is dominated by the feature related to the 3D phase (Figure 5b) within Δ*t* = 1 ps after excitation.^34,45^ This behavior results from a combination of direct absorption by the 3D perovskite as observed immediately after photoexcitation (Δ*t* = 1 ps) as well as charge transfer from lower-dimensional phases (Figure S11, Supporting Information) at longer delay times up to Δ*t* = 200 ps, followed by subsequent decay.

In contrast, a coevaporated film deposited on bare ITO (0 mM PPAc) shows a strong photobleach at 570 nm corresponding to the *n* = 2 phase (Figure 5c,d). This agrees with observations made with UV–vis–NIR, XRD, and GIWAXS measurements that show the *n* = 2 phase to be dominant in coevaporated films (Figures 1c,e and 3b). However, the spectral lineshapes also show the presence of additional photobleaching features in the 600–750 nm range that appear at Δ*t* = 0.5 ps after excitation. These features are related to Ruddlesden–Popper structures with higher *n*-values (*n* = 3–5), as observed in UV–vis–NIR spectra (Figure 3b), which quench charge-carriers from the *n* = 2 phase. However, it is likely that these low-energy phases represent a small volume fraction of the film and therefore do not significantly contribute to charge-carrier dynamics. Their absence in structural characterization data may also be related to their low crystallinity and further emphasizes the efficacy of pump–probe spectroscopy in observing secondary phases in Ruddlesden–Popper films.^22,34^

Upon increasing the PPAc concentration (1 mM), the photobleaching feature related to *n* = 2 is retained while secondary features (*n* = 3 and *n* = 4) diminish and are visible as distinct minima (Figure 5e,f). Finally, at high PPAc concentration (20 mM), an additional feature related to the *n* = 1 phase appears at λ ∼ 520 nm (Figure 5g,h) and features related to secondary phases (*n* > 2) are considerably suppressed (Figure S12, Supporting Information). This agrees with the hypothesis that PPAc-templated crystallization of the Ruddlesden–Popper film shifts the phase distribution to lower *n*-valued structures, as evidenced through absorption and structural characterization (Figure 3). Here, it must be noted that the population of the *n* = 2 phase is delayed with increasing proportion of the *n* = 1 phase (increasing PPAc concentration), indicating a possible charge transfer contribution to the population alongside the direct absorption of the *n* = 2 phase (Figure S13, Supporting Information). It is also remarkable that the *n* = 2 phase contributes significantly to charge-carrier dynamics even though the structural behavior (Figure 3d,h) indicates a relatively smaller proportion compared to the *n* = 1 phase.

Taken together, these observations show that coevaporation and the use of a PPAc templating agent impact charge-carrier dynamics of Ruddlesden–Popper films. First, the phase purity achieved due to coevaporation increases the contribution of the *n* = 2 phase in charge-carrier behavior compared to other higher-dimensional (*n* > 2) phases. Second, a stochastic distribution of higher-dimensional (*n* = 3 – 5) structures is shown to play a role in charge-carrier dynamics in coevaporated films. It must nevertheless be noted that these structures participate insignificantly in coevaporated films compared to solution-processed films (Figures 5a,b). Finally, the progressive inclusion of PPAc causes the phase distribution to shift from higher dimensionality to lower dimensionality, which minimizes charge-carrier quenching by lower-energy phases.

## Conclusions

In this work, we have demonstrated the use of thermal coevaporation for the growth of oriented Ruddlesden–Popper thin films with high phase purity. We achieve that by eliminating interactions between precursors and solvents that undermine phase purity in solution-processed films and develop homogeneous quasi-2D (*n* = 2) coevaporated films. We then demonstrate how the uptake of different organic precursors in the coevaporated film can be modulated using substrate modification strategies. We use a phosphonic acid-based surface modifier (PPAc) that interacts strongly with PEA^+^ compared with FA^+^, thereby favoring the crystallization of 2D (*n* = 1) structures instead of quasi-2D (*n* = 2) structures. This results in a shift in the phase distribution to lower *n*-values as a function of the increasing PPAc concentration. As a result, we show the growth of 2D/quasi-2D heterostructure films where the PPAc concentration and the Ruddlesden–Popper film thickness can be used to modulate the volume fractions of different structural phases. Taken together, our results introduce new processing routes for the growth of tunable, phase-pure Ruddlesden–Popper structures, which can be used in efficient optoelectronic applications. It overcomes a key bottleneck of uncontrolled growth of large volumes of undesired low-energy phases, allowing a greater contribution from quasi-2D phases in charge-carrier dynamics.

However, despite the significantly higher purity of the quasi-2D phase achieved by coevaporation, ultrafast pump–probe spectroscopy also reveals a small minority of undesired secondary phases (*n* > 2), underlining important challenges in suppressing their crystallization in coevaporated films. To that effect, stabilization of the quasi-2D phase can likely be achieved by engineering the formation dynamics of desired Ruddlesden–Popper phases or through process optimization to minimize secondary phase crystallization.^6,29,41−45^ Furthermore, this work also highlights the sensitivity of thermal coevaporation processes to precursor uptake and the role that substrates play in altering the adsorption of organic precursors and subsequently controlling quasi-2D film crystallization.^44−47^ We speculate that further study of interactions between organic precursors and surface modifiers will allow improved control of phase selectivity and phase distribution, enabling deposition of complex heterostructures and vapor-processed thin film-based devices. Specifically, the interactions between evaporated precursors and a phosphonic acid-based surface modifier that we identify in this work may also be applicable to several commonly used passivating agents and self-assembled charge-transporting monolayers used for photovoltaics, light emission, and photodetection that use phosphonic or carboxylic acid-based anchoring groups to bind to metal oxide substrate surfaces.^48−53^ Among them, several phosphonic acid-based hole-transport molecules have also been successfully coated using thermal evaporation, emphasizing their compatibility with vapor-processed devices.^54^ As a result, such molecules may serve the dual purpose of participating in Ruddlesden–Popper crystallization as well as charge transport. Additional efforts to reduce deposition times while maintaining film quality would also benefit device implementation and throughput.^55,56^ Finally, beyond the *n* = 1 and *n* = 2 phases demonstrated in this work, dimensional tunability through a combination of process optimization and the development of new functionalizing molecules can also expand the structural range of evaporated Ruddlesden–Popper films, enabling applications such as color-tunability in light-emitting diodes and photodetectors, and allow the fabrication of stable Ruddlesden–Popper wide-bandgap (2 ≤ *n* ≤ 5 for approximately 2.0–1.8 eV bandgap) top-cells for multijunction photovoltaics.^2,57,58^ Here, the impact of film composition and functionalized interfaces on structural orientation is especially important to tune in- and out-of-plane charge transport and, in addition to the scalability of device platforms, will be an important area of research to advance evaporated quasi-2D-based electronics.^9,11,47,59,60^

## Supporting Information Available

Experimental section with details about sample preparation, characterization and DFT calculations; additional figures with *ex situ* and *in situ* structural (GIWAXS) characterization, profilometry measurements, and ultrafast pump–probe spectroscopy of Ruddlesden–Popper films (PDF)
