# Supplementary material for: Dimensional Control in Phase-Pure Coevaporated Quasi-2D Ruddlesden–Popper Structures
Source: J Am Chem Soc. 2025 Apr 29;147(19):16119–28. doi: 10.1021/jacs.4c18641 (PMC12082631; doi:10.1021/jacs.4c18641)
Supplement: Supplementary file 1 — ja4c18641_si_001.pdf [file ja4c18641_si_001.pdf]

Supporting Information

Dimensional Control in Phase-Pure Co-Evaporated Quasi-2D Ruddlesden-Popper Structures

Kunal Datta[1,$], Pranav Khadilkar[1], Honghu Zhang[2], Diana K. LaFollette[1], Esteban Rojas-Gatjens[3,#], Ruipeng Li[2], Guoxiang Hu[1,3], Juan-Pablo Correa-Baena[1,3]

[1] School of Materials Science and Engineering, Georgia Institute of Technology, Atlanta, Georgia 30332, United States of America

[2] National Synchrotron Light Source II, Brookhaven National Laboratory, Upton, New York 11973, United States of America

[3] School of Chemistry and Biochemistry, Georgia Institute of Technology, Atlanta, Georgia 30332, United States of America

Present Address:

[$] K.D.: Molecular Materials and Nanosystems, Eindhoven University of Technology, P.O. Box 513, 5600 MB, Eindhoven, The Netherlands

[#] E.R.-G: Department of Chemistry, Columbia University, New York, New York 10027, United States of America

EXPERIMENTAL SECTION

Sample Preparation

Propylphosphonic acid stock solution (20 mM) was prepared by dissolving propylphosphonic acid in ethanol (Sigma Aldrich, 99 %) and sonicating for 15 min. and subsequently diluted with anhydrous ethanol to achieve the required precursor concentration. $PEA_2FAPb_2I_7$ solution was prepared by

dissolving stoichiometric amounts of PEAI (Greatcell Solar), PbI_2 (TCI, > 99.99 %) and FAI (Greatcell Solar) precursors in DMF (Sigma Aldrich) in an N_2–containing glovebox.

1 in. x 1 in. patterned glass|ITO substrates were cleaned by sonication 15 min in a 2% Hellmanex solution, followed by 10 min sequential steps in deionized water, acetone, and isopropanol. The substrates were then treated with UV-ozone and immediately transferred to an N_2–containing glovebox. The phosphonic acid solution was cast onto the substrate and coated using spin-coating at 3000 rpm for 30 s followed by annealing at 100 °C for 10 min. The substrates were then transferred to the evaporator chamber for the deposition of the Ruddlesden-Popper layer. Thermal evaporation was conducted using a three-source deposition process with PbI_2 (Sigma Aldrich, 99.999 %), PEAI (Greatcell Solar) and FAI (Greatcell Solar) precursor powders. The temperatures of the sources were first ramped to 270 °C, 120 °C and 140 °C, respectively. Upon achieving stable rates of 0.24 Å/s, 0.40 Å/s and 0.095 Å/s respectively, the sample shutter was opened, and the substrates were exposed to the evaporation cone. The film thickness was monitored using a quartz crystal microbalance placed near the substrate stage. The samples were not annealed following the evaporation process. Solution-processing of $PEA_2FAPb_2I_7$ was conducted by casting the precursor solution and spin-coating at 4000 rpm for 45 s followed by annealing at 100 °C for 30 min.

Characterization

XRD measurements were performed using a third-generation Panalytical Empyrean diffractometer. GIWAXS measurements were conducted at the 11-BM Complex Materials Scattering (CMS) beamline at the National Synchrotron Light Source II at Brookhaven National Laboratory, Upton, New York. The films were reduced to a size of approx. 5 mm × 5 mm. 2D images were acquired at X-ray incidence angles of 0.05° and 0.5° with an acquisition time of 10 s, using an X-ray beam energy of 13.5 keV, and 0.2 mm (height) × 0.05 mm (width) size. Beam divergence was 0.1 mrad and energy resolution 0.7%. In-situ characterization was conducted using a controlled relative humidity chamber where dry

air was fed into a water bubbler to achieve at a relative humidity of 100% and was then fed into the sample chamber.[1] Data analysis was performed building on the SciAnalysis package provided at the beamline. Out-of-plane sectors were taken between – 10° and + 10° from the substrate normal (polar angle (χ) = 0°). Peaks were fit with pseudo-Voigt lineshapes with equal Gaussian and Lorentzian contributions (shape factor 1) using the Multi-Peak Fit module of Igor Pro.[2] UV-vis-NIR spectra were acquired using Cary 5000 UV-vis/NIR spectrometer. Film thickness was measured using a Bruker Dektak XT profilometer. Transient absorption spectroscopy was conducted using an ultrafast laser system (Pharos Model PH1-20-0200-02-10, Light Conversion) with output pulses of 1030nm at 100 kHz, with a power of 20 W and a pulse duration of 220 fs. The measurements were carried out in a commercial set-up (Light Conversion HERA). The pump energy 2.64 eV was generated by feeding 10 W from the laser output to a commercial optical parametric amplifier (Orpheus, Light Conversion) while a small portion was focused onto a sapphire crystal to obtain a single-filament white light continuum covering the spectral range 490 – 1050 nm for the probe beam. The data was generated after averaging over five sequential measurements.

DFT calculations

Spin-polarized density functional theory (DFT) calculations were performed using the Vienna ab initio simulations package (VASP).[3,4] The electron exchange-correlation was represented by the functional of Perdew, Burke and Ernzerhof (PBE) of generalized gradient approximation (GGA).[5] The ion-electron interaction was described with the projector augmented wave (PAW) method.[6] A cutoff energy of 400 eV was used for the plane-wave basis set. The molecules were added to a cubic box (30 Å × 30 Å × 30 Å), and the Γ point only was used to sample the k space. Geometry optimizations were performed using the conjugate gradient algorithm with the criterion that all the residual force components on each atom be <0.03 eV/Å. Van der Waals interactions were also included using the DFT-D3 method.[7]

ADDITIONAL FIGURES

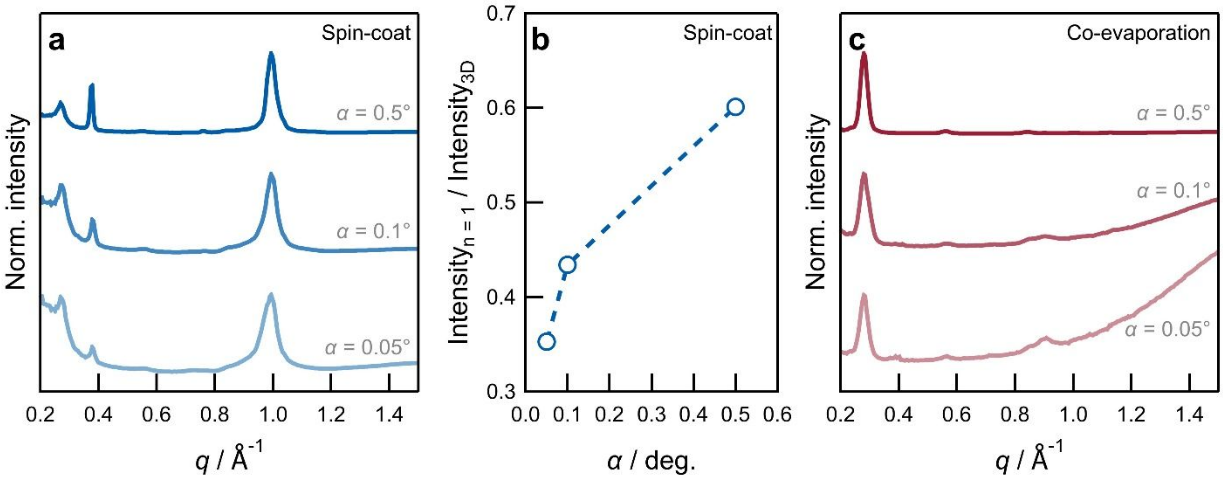

Figure S1. (a) Angle-dependent GIWAXS sector averages of spin-coated $PEA_2FAPb_2I_7$ film. (b) Intensity ratios of peaks from panel (a) corresponding to n = 1 phase and 3D phase. (c) Angle-dependent GIWAXS sector averages of co-evaporated $PEA_2FAPb_2I_7$ film. The data is normalized to the maximum intensities of the peak related to the 3D phase (q ~ 0.99 $Å^{-1}$) in panel (a) and to the peak related to the n = 2 phase (q ~ 0.28 $Å^{-1}$) in panel (c).

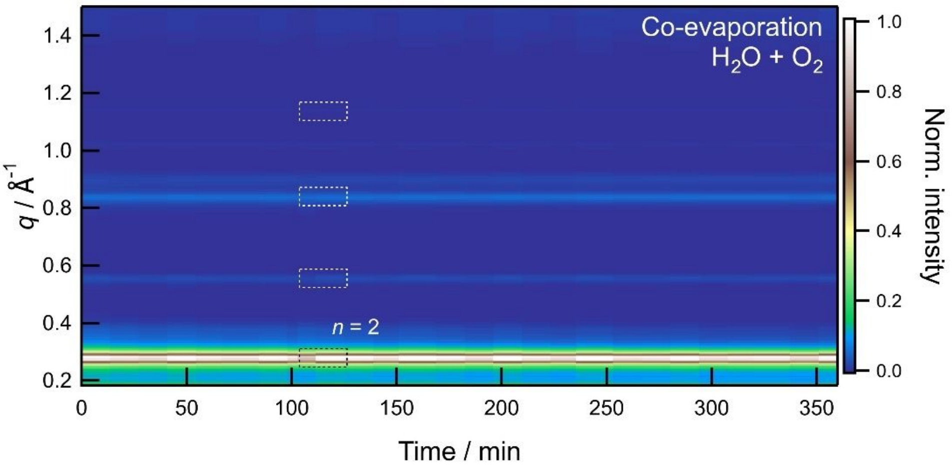

Figure S2. 2D map of GIWAXS circular average as a function of time for 150 nm thick co-evaporated $n = 2$ film exposed to humidity and oxygen environment (RH ~ 100%). Dashed boxes represent peaks related to the $n = 2$ phase. The data is normalized to the intensity of the diffraction feature related to the $n = 2$ phase ($q \sim 0.28$ Å^{-1}) at the start of the measurement.

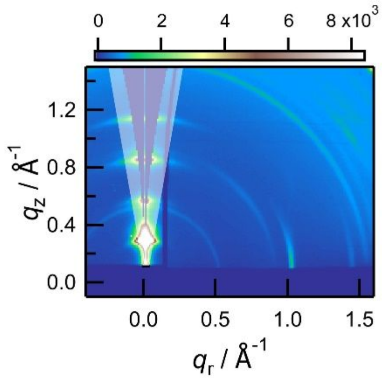

Figure S3. Schematic of region (shaded) used to conduct sector average analysis. The data shown is the 2D GIWAXS pattern of a co-evaporated Ruddlesden-Popper film coated on a PPAc-functionalized substrate with 1 mM PPAc concentration, as shown in Figure 3e.

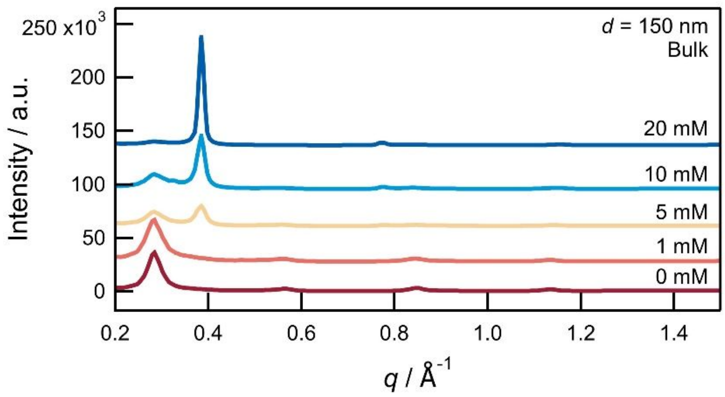

Figure S4. Sector average of GIWAXS patterns of 150 nm thick Ruddlesden-Popper films coated on PPAc-functionalized substrates with different PPAc concentrations, acquired at an incidence angle α = 0.5° (Bulk).

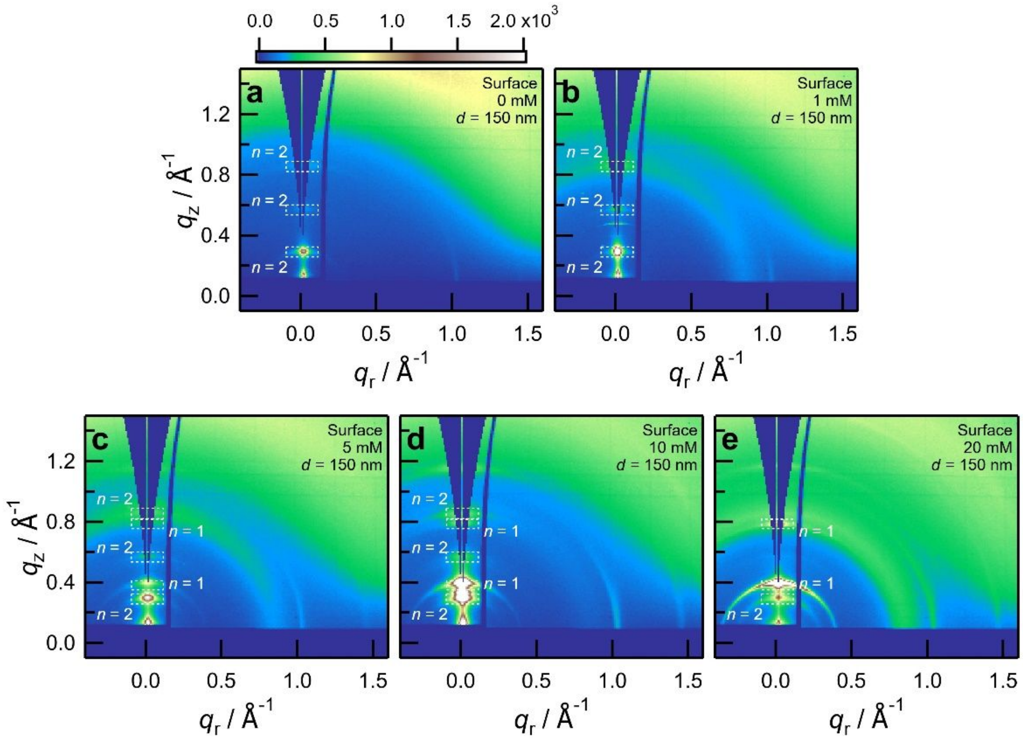

Figure S5. 2D GIWAXS patterns acquired with α = 0.05° (Surface) of 150 nm thick co-evaporated quasi-2D Ruddlesden-Popper films coated on PPAc functionalized substrates with PPAc concentrations (a) 0 mM, (b) 1 mM, (c) 5 mM, (d) 10 mM and (e) 20 mM.

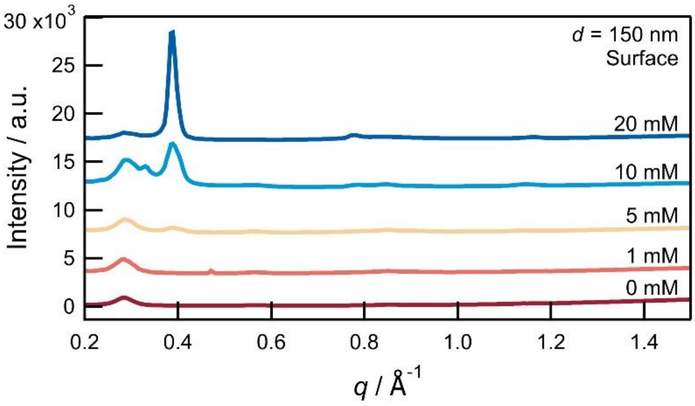

Figure S6. Sector average of GIWAXS patterns of 150 nm thick Ruddlesden-Popper films coated on PPAc-functionalized substrates with different PPAc concentrations (0 – 20 mM), acquired at an incidence angle α = 0.05° (Surface).

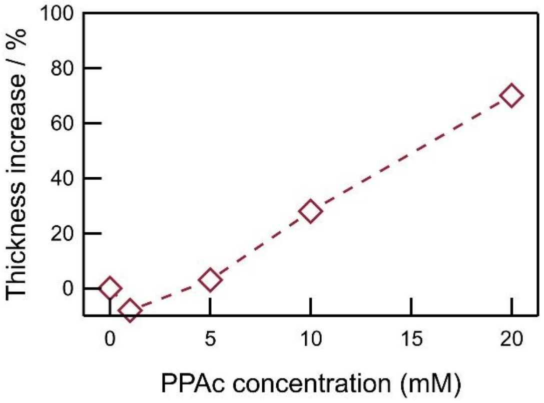

Figure S7. Change in Ruddlesden-Popper film thickness as a function of PPAc concentration.

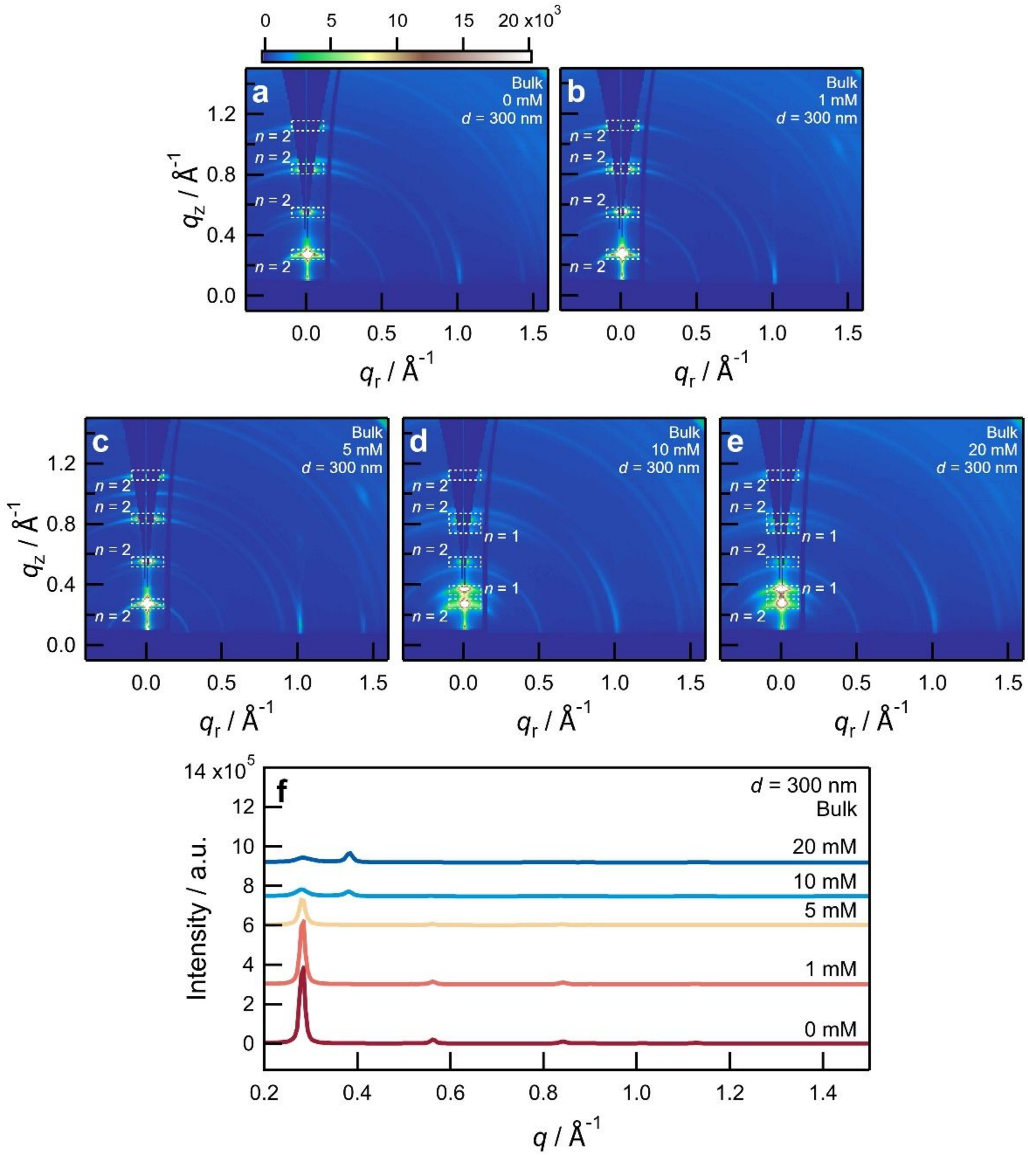

Figure S8. (a – e) 2D GIWAXS patterns and (f) corresponding sector averages acquired with α = 0.5° (Bulk) of 300 nm thick co-evaporated quasi-2D Ruddlesden-Popper films coated on PPAc functionalized substrates with PPAc concentrations (a) 0 mM, (b) 1 mM, (c) 5 mM, (d) 10 mM and (e) 20 mM.

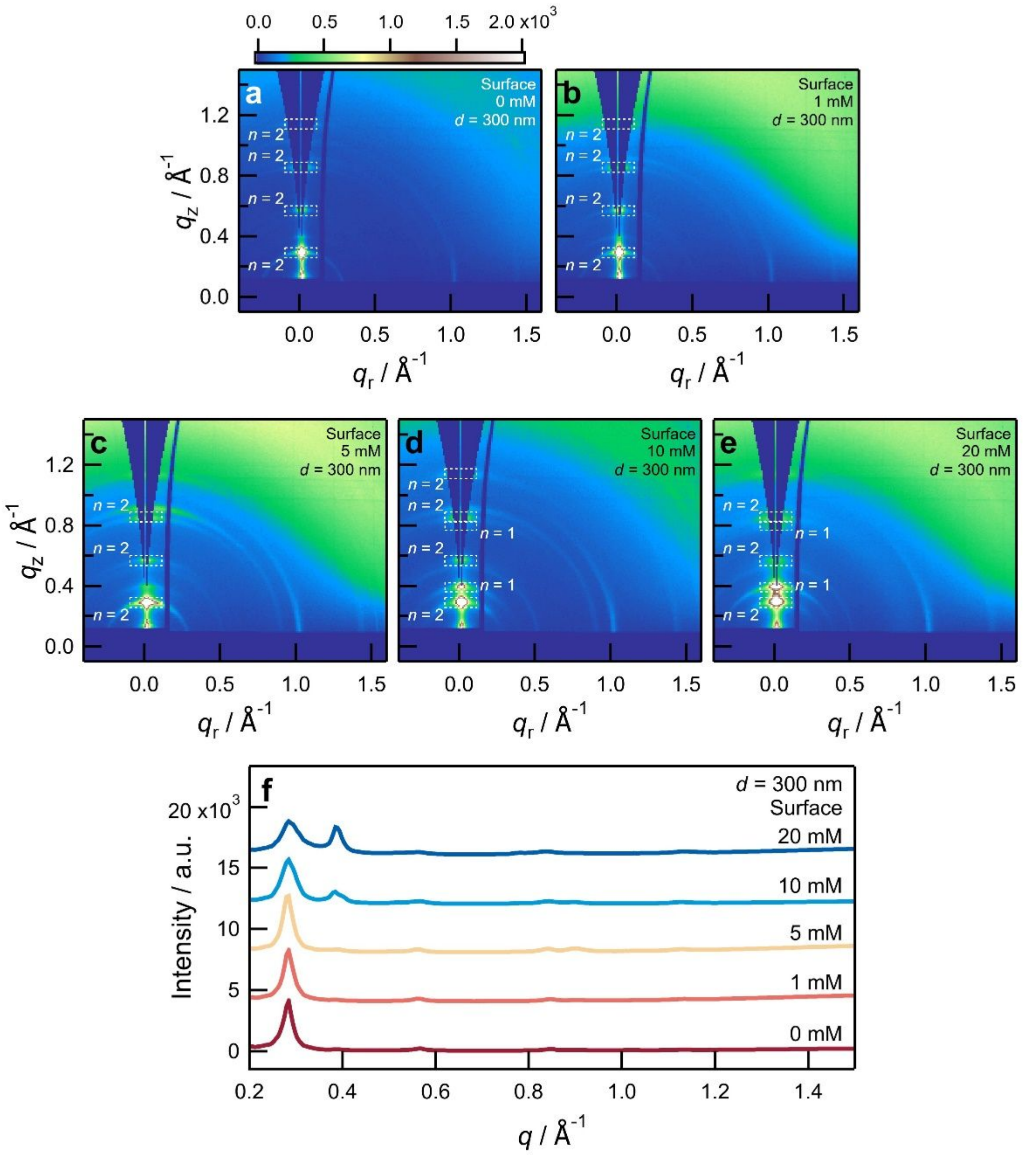

Figure S9. (a – e) 2D GIWAXS patterns and (f) corresponding sector averages acquired with α = 0.05° (Surface) of 300 nm thick co-evaporated quasi-2D Ruddlesden-Popper films coated on PPAc functionalized substrates with PPAc concentrations (a) 0 mM, (b) 1 mM, (c) 5 mM, (d) 10 mM and (e) 20 mM.

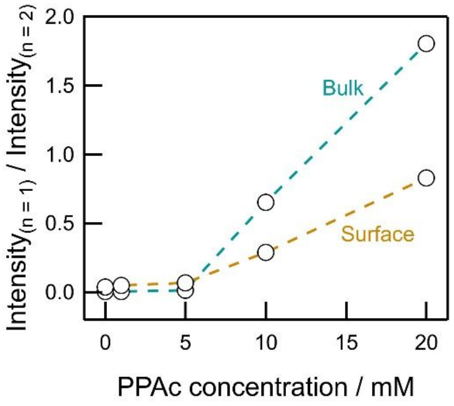

Figure S10. Intensity ratio of $n = 1$ and $n = 2$ diffraction features of 300 nm thick Ruddlesden-Popper films acquired at $\alpha = 0.05°$ (Surface, ochre) and $\alpha = 0.5°$ (Bulk, teal) as function of PPAc concentration.

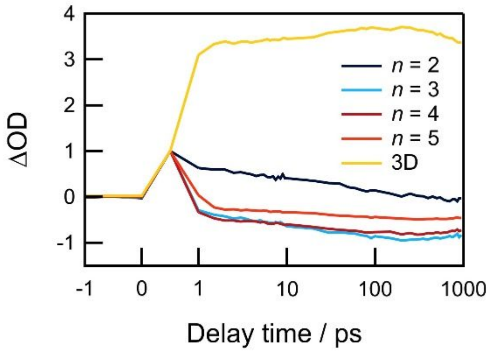

Figure S11. Transient spectra acquired from ultrafast pump-probe spectroscopy of spin-coated Ruddlesden-Popper film showing transient behavior of different structural phases. The traces are normalized to the intensity at 0.5 ps delay time after photoexcitation by pump beam. Note that the x-axis is plotted on the linear scale between – 1 and 1 ps and on the logarithmic scale between 1 and 1000 ps.

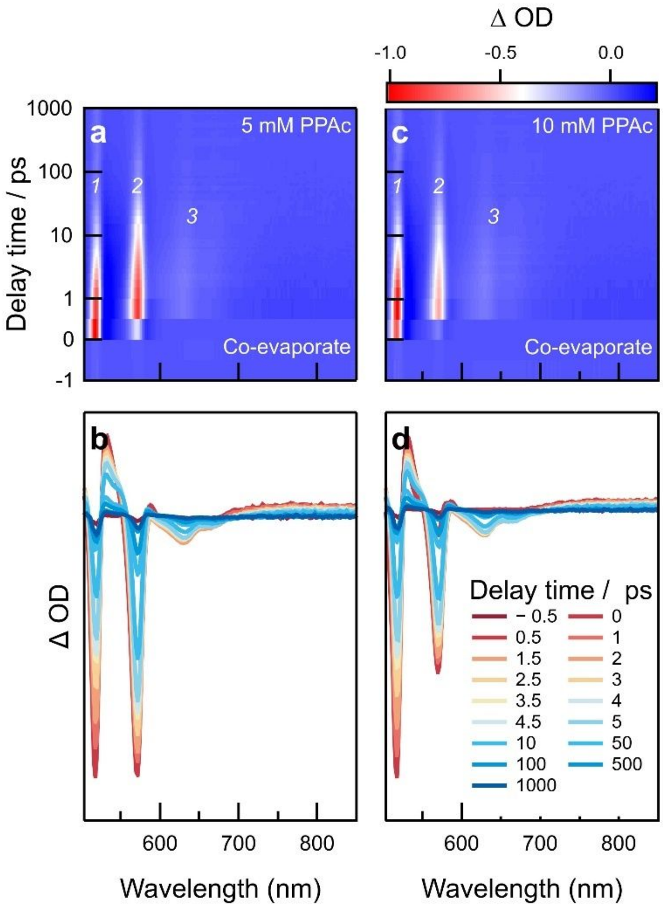

Figure S12. Transient absorption spectroscopy of co-evaporated Ruddlesden-Popper films. 2D heatmaps and linecuts of spin-coated film coated on PPAc functionalized substrates with PPAc concentration (a,b) 5 mM and (c,d) 10 mM. Note that the y-axis is plotted on the linear scale between – 1 and 1 ps delay time and on the log scale between 1 and 1000 ps delay time in panels (a) and (c).

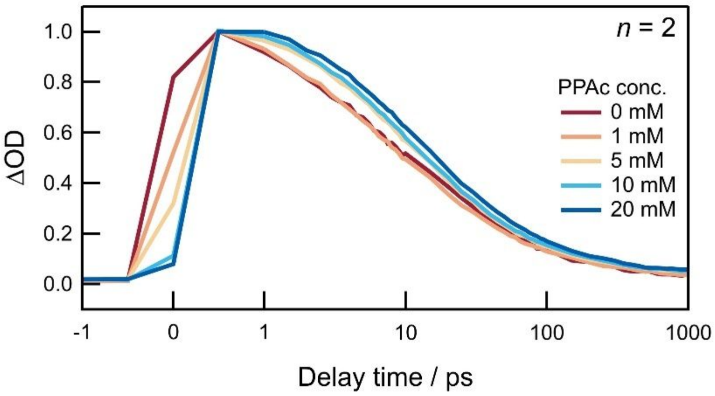

Figure S13. Transient spectra acquired from ultrafast pump-probe spectroscopy of 150 nm-thick co-evaporated Ruddlesden-Popper film showing transient behavior of the n = 2 phase. The films are coated on PPAc functionalized substrates with PPAc concentration between 0 and 20 mM in the precursor solution. The traces are normalized to the intensity at 0.5 ps delay time after photoexcitation by pump beam. Note that the x-axis is plotted on the linear scale between – 1 and 1 ps and on the logarithmic scale between 1 and 1000 ps.

REFERENCES

(1) Hidalgo, J.; Kaiser, W.; An, Y.; Li, R.; Oh, Z.; Castro-Méndez, A.-F.; LaFollette, D. K.; Kim, S.; Lai, B.; Breternitz, J.; Schorr, S.; Perini, C. A. R.; Mosconi, E.; De Angelis, F.; Correa-Baena, J.-P. Synergistic Role of Water and Oxygen Leads to Degradation in Formamidinium-Based Halide Perovskites. *J. Am. Chem. Soc.* **2023**, *145* (45), 24549–24557. https://doi.org/10.1021/jacs.3c05657.

(2) Kennard, R. M.; Dahlman, C. J.; Chung, J.; Cotts, B. L.; Mikhailovsky, A. A.; Mao, L.; DeCrescent, R. A.; Stone, K. H.; Venkatesan, N. R.; Mohtashami, Y.; Assadi, S.; Salleo, A.; Schuller, J. A.; Seshadri, R.; Chabinyc, M. L. Growth-Controlled Broad Emission in Phase-Pure Two-Dimensional Hybrid Perovskite Films. *Chem. Mater.* **2021**, *33* (18), 7290–7300. https://doi.org/10.1021/acs.chemmater.1c01641.

(3) Kresse, G.; Furthmüller, J. Efficient Iterative Schemes for Ab Initio Total-Energy Calculations Using a Plane-Wave Basis Set. *Phys. Rev. B* **1996**, *54* (16), 11169–11186. https://doi.org/10.1103/PhysRevB.54.11169.

(4) Kresse, G.; Furthmüller, J. Efficiency of Ab-Initio Total Energy Calculations for Metals and Semiconductors Using a Plane-Wave Basis Set. *Computational Materials Science* **1996**, *6* (1), 15–50. https://doi.org/10.1016/0927-0256(96)00008-0.

(5) Perdew, J. P.; Burke, K.; Ernzerhof, M. Generalized Gradient Approximation Made Simple. *Phys. Rev. Lett.* **1996**, *77* (18), 3865–3868. https://doi.org/10.1103/PhysRevLett.77.3865.

(6) Blöchl, P. E. Projector Augmented-Wave Method. *Phys. Rev. B* **1994**, *50* (24), 17953–17979. https://doi.org/10.1103/PhysRevB.50.17953.

(7) Grimme, S.; Antony, J.; Ehrlich, S.; Krieg, H. A Consistent and Accurate Ab Initio Parametrization of Density Functional Dispersion Correction (DFT-D) for the 94 Elements H-Pu. *The Journal of Chemical Physics* **2010**, *132* (15), 154104. https://doi.org/10.1063/1.3382344.
